# Androgen Suppresses the Proliferation of Androgen Receptor-Positive Castration-Resistant Prostate Cancer Cells via Inhibition of Cdk2, CyclinA, and Skp2

**DOI:** 10.1371/journal.pone.0109170

**Published:** 2014-10-01

**Authors:** John M. Kokontis, Hui-Ping Lin, Shih Sheng Jiang, Ching-Yu Lin, Junichi Fukuchi, Richard A. Hiipakka, Chi-Jung Chung, Tzu-Min Chan, Shutsung Liao, Chung-Ho Chang, Chih-Pin Chuu

**Affiliations:** 1 The Ben May Department for Cancer Research, The University of Chicago, Chicago, Illinois, United States of America; 2 National Institute of Cancer Research, National Health Research Institutes, Miaoli County, Taiwan; 3 Institute of Cellular and System Medicine, National Health Research Institutes, Miaoli, Taiwan; 4 Pharmaceuticals and Medical Devises Agency, Tokyo, Japan; 5 Department of Health Risk Management, China Medical University, Taichung City, Taiwan; 6 Department of Medical Education and Research, China Medical University Beigang Hospital, Yunlin, Taiwan; 7 Department of Medical Education and Research, Tainan Municipal An-Nan Hospital-China Medical University, Tainan, Taiwan; 8 Graduate Institute of Basic Medical Science, China Medical University, Taichung City, Taiwan; 9 Biotechnology Center, National Chung Hsing University, Taichung, Taiwan, Taichung City, Taiwan; 10 Ph.D. program in Environmental and Occupational Medicine, Kaohsiung Medical University, Kaohsiung City, Taiwan; UC Davis Comprehensive Cancer Center, United States of America

## Abstract

The majority of prostate cancer (PCa) patient receiving androgen ablation therapy eventually develop castration-resistant prostate cancer (CRPC). We previously reported that androgen treatment suppresses Skp2 and c-Myc through androgen receptor (AR) and induced G1 cell cycle arrest in androgen-independent LNCaP 104-R2 cells, a late stage CRPC cell line model. However, the mechanism of androgenic regulation of Skp2 in CRPC cells was not fully understood. In this study, we investigated the androgenic regulation of Skp2 in two AR-positive CRPC cell line models, the LNCaP 104-R1 and PC-3^AR^ Cells. The former one is an early stage androgen-independent LNCaP cells, while the later one is PC-3 cells re-expressing either wild type AR or mutant LNCaP AR. Proliferation of LNCaP 104-R1 and PC-3^AR^ cells is not dependent on but is suppressed by androgen. We observed in this study that androgen treatment reduced protein expression of Cdk2, Cdk7, Cyclin A, cyclin H, Skp2, c-Myc, and E2F-1; lessened phosphorylation of Thr14, Tyr15, and Thr160 on Cdk2; decreased activity of Cdk2; induced protein level of p27^Kip1^; and caused G1 cell cycle arrest in LNCaP 104-R1 cells and PC-3^AR^ cells. Overexpression of Skp2 protein in LNCaP 104-R1 or PC-3^AR^ cells partially blocked accumulation of p27^Kip1^ and increased Cdk2 activity under androgen treatment, which partially blocked the androgenic suppressive effects on proliferation and cell cycle. Analyzing on-line gene array data of 214 normal and PCa samples indicated that gene expression of Skp2, Cdk2, and cyclin A positively correlates to each other, while Cdk7 negatively correlates to these genes. These observations suggested that androgen suppresses the proliferation of CRPC cells partially through inhibition of Cyclin A, Cdk2, and Skp2.

## Introduction

In 1941, Charles Huggins reported that androgen ablation therapy caused regression of primary and metastatic androgen-dependent prostate cancer (PCa) [1]. Androgen ablation therapy, using luteinizing hormone-releasing hormone agonists (LH-RH) or bilateral orchiectomy, has become a primary treatment for metastatic prostate cancer [2]. The majority of patients experience an initial rapid decline in PSA followed by a slower decline to the nadir [2]. However, 80–90% of the patients eventually develop castration-resistant prostate cancer (CRPC) 12–33 months after androgen ablation therapy with a median overall survival of 12–24 months [3]. Androgen receptor (AR) plays important role in the development, progression, and metastasis of prostate cancer [4]. Increase in AR mRNA and protein is observed in CRPC tumors compared to the primary prostate tumors [5], [6].

LNCaP is a commonly used cell line established from a human lymph node metastatic lesion of prostatic adenocarcinoma. LNCaP cells express androgen receptor (AR) and prostate specific antigen (PSA) [7], [8]. Previously, we developed a PCa progression model using LNCaP cells. Androgen-dependent LNCaP 104-S cells were cultured in androgen-depleted conditions to mimic patients receiving androgen ablation therapy [9]–[11]. A small population of castration-resistant cells named LNCaP 104-R1 emerged after 10 months [9]–[11]. After additional 8 months culturing in androgen-depleted medium, LNCaP 104-R1 cells gave rise to LNCaP 104-R2 cells, which proliferated much faster than 104-R1 cells [10]. Proliferation of LNCaP 104-R1 and 104-R2 cells is androgen-independent but is suppressed by physiological concentrations of androgen [9], [10], [12], [13]. LNCaP 104-R1 and 104-R2 cells mimic early and late CRPC cells, respectively [14]. Following androgen treatment, the majorities of LNCaP 104-R1 and 104-R2 cells underwent G1 cell cells arrest and died eventually with only a small population of cells survived and resumed growing, named R1Ad [10] and R2Ad [15], respectively. However, proliferation of R1Ad cells is androgen-dependent and can be controlled by androgen ablation therapy [12], while proliferation of R2Ad cells is androgen-insensitive and does not respond to further hormone therapy [15]. Therefore, patient with early stage CRPC tumors may benefit from androgen treatment. We previously reported that androgen treatment suppresses S-phase kinase-associated protein 2 (Skp2) and c-Myc through AR in LNCaP 104-R2 cells, thus inducing G1 cell cycle arrest and growth inhibition [15]. Oncogenic activity and androgenic regulation of c-Myc have been studied intensively. However, androgenic regulation of Skp2 in CRPC cells is less understood.

Skp2, an F-box protein, and its cofactor Cks1 are the substrate-targeting subunits of the SCF (Skp1/Cul1/F-box protein) ubiquitin ligase complex. SCF is an E3 ubiquitin ligase complex which regulates the S phase entry of cells by inducing the degradation of the cyclin-dependent kinase inhibitors p21^Cip1^ and p27^Kip1^ [16], [17]. Skp2 targets p27^Kip1^ by phosphorylating p27^Kip1^ at T187 for ubiquitination and degradation [18]–[20]. Skp2 forms a stable complex with the cyclin A-cyclin-dependent kinase 2 (Cdk2) [20]. Skp2 is phosphorylated by Cdk2 at Ser64 [20] and by Akt at Ser72 [21]. Phosphorylation of Ser64 and Ser72 on Skp2 contributes to the stabilization of Skp2 by preventing its association with APC/CCdh1 [17], [18], [20], [21]. Both luminal and basal epithelial cells in normal prostate exhibit very low Skp2 levels, however, Skp2 levels increase dramatically in both prostatic intraepithelial neoplasm (PIN) and PCa [22], [23]. Up-regulation of Skp2 correlates to lower p27^Kip^ expression, higher Gleason score, and more advanced pathological stage of PCa [22], [24]. Up-regulation of Skp2 in PCa is also independently associated with a higher risk of PCa recurrence after surgery [22], [24]. Skp2 overexpression in PCa cells stimulates PCa cell proliferation and increases the tumorigenesis in xenograft tumor model [25].

Cdk2 is a member of the cyclin-dependent kinase family of Ser/Thr protein kinases [26]. Complex of Cdk2-cyclin E is required for the transition of cells from G1 to S phase, while binding between Cdk2 and cyclin A is required to progress through the S phase [26]. Activation of Cdk2 complexes requires dephosphorylation of Thr14 and Tyr15 on Cdk2 by cdc25 phosphatase and phosphorylation of Thr160 on Cdk2 [26], [27], which is mediated by CAK, a complex of Cdk7 and cyclin H [28]. Cyclin A is a member of the cyclin family, a group of proteins that function in regulating progression through the cell cycle. Transcription of cyclin A is tightly regulated and synchronized with cell cycle progression by the transcription factor E2F in a negative feedback loop [29].

LNCaP cells express a mutant AR (T877A) that displays relaxed ligand binding specificity [30], [31], we thus generated AR-positive PC-3 cells by overexpressing either wild type AR (PC-3^AR^) or LNCaP mutant AR (PC-3^LNCaP-AR^) in AR-negative PC-3 cells as other CRPC cell liens. We used LNCaP 104-R1 cells, a model mimics early stage CRPC, as well as PC-3^AR^ and PC-3^LNCaP-AR^ cells to examine the androgenic regulation of Skp2 and related Cyclin-dependent kinases (Cdks) as well as cell proliferation in these CRPC cells.

## Materials and Methods

### Materials

Synthetic androgen R1881 and antiandrogen Casodex (bicalutamide) were obtained from Perkin Elmer (Boston, MA, U.S.A.) and Astrazeneca (Wilmington, DE, U.S.A.), respectively. [a-^32^P]dCTP (3000 Ci/mmole) and [g-^32^P]ATP (5000 Ci/mmole) were from Amersham (Arlington Heights, IL, U.S.A.). Peptides were synthesized by the University of Chicago Cancer Research Center oligopeptide synthesis facility.

### Cell culture

LNCaP 104-R1 cells and PC-3 sublines were gifts from Dr. Shutsung Liao (The University of Chicago, IL, U.S.A.). LNCaP 104-R1 cells were derived from parental androgen-dependent LNCaP 104-S cells, which were generated from LNCaP FGC clone (ATCC CRL-1740). The LNCaP 104-R1 cells and PC-3 sublines have been described in previous publications [10]–[13], [15], [32]–[34]. LNCaP 104-R1, PC-3, PC-3^AR^, and PC-3^LNCaPAR^ cells were maintain in DMEM with 10% charcoal-stripped FBS (CS-FBS).

### Western blotting analysis

LNCaP 104-R1 cells or PC-3 sublines were washed with PBS and lysed in 2× Laemmli buffer without bromophenol blue dye. Protein concentration of the cell lysates was determined with the Bradford reagent (Bio-Rad Laboratories, Hercules, CA, U.S.A.). Antibody for Skp2 and p21^cip1/waf1^ were from Santa Cruz Biotechnology (Santa Cruz, CA, U.S.A.). Antibodies for cyclin E, Cdk2, and phospho-Cdk2 Thr160 were from Cell Signaling (Danvers, MA, U.S.A.). Cyclin A and E2F-1 antibodies were from Millipore (Billerica, MA, U.S.A.). The p27^Kip1^ antibody was from BD Transduction Laboratories (Lexington, KY, U.S.A.). The phospho-Cdk2 Tyr15 and phospho-Cdk2 Thr14 antibodies were purchased from Epitomics (Burlingame, CA, U.S.A.). Cdk7 and Cyclin H were from Abnova (Taipei, Taiwan). Detection of α-tubulin (Sigma, St. Louis, MO, U.S.A.) or β-actin (Novus, Littleton, CO, U.S.A.) was used as the loading control. For SDS-PAGE of Cdk2, adjustment of the pH of the separating gel buffer to 8.5 was required for resolution of the faster-migrating isoform. Intensity of bands for different proteins was quantified with EPSON stylus TX130 using UN-SCAN-IT gel 6.1 software.

### Cell proliferation assay

LNCaP 104-R1 cells or PC-3 sublines were seeded at a density of 3×10^3^ cells/well in 96-well plates with 100 µl DMEM medium containing 10% CS-FBS. Proliferation assays were performed as described previously [13], [15], [33]–[39]. All readouts were normalized to the average of the control condition in each individual experiment. The experiment was repeated three times. Ten wells were used for each condition. The mean and standard deviation represented the average and standard deviation respectively of the results from all 30 wells in the three experiments.

### Flow Cytometric Analysis

Cells were seeded at the density of 5×10^5^ cells in 6-cm dishes. Flow cytometric assay was performed as previously described [13], [15], [35]–[37], [39].

### Real-Time Quantitative Polymerase Chain Reaction

Total RNA was isolated using the TriZol Reagent (Invitrogen, Carlsbad, CA) and was treated with DNase I (DNA-free; Ambion, Austin, TX). Reverse transcription was performed with random hexamers and Moloney murine leukemia virus reverse transcriptase (Omniscript; Qiagen, Valencia, CA). The TaqMan primer/probe was designed using Primer Express (Applied Biosystems, Foster City, CA). The 5′ end of the probe was labeled with reporter-fluorescent dye FAM. The 3′ end of probe was labeled with quencher dye TAMRA. The sequences of CDKN1B primers, 5′-CCGGTGGACCACGAAGAGT-3′ and 5′-GCTCGCCTCTTCCATGTCTC-3′; CDKN1B probe, 5′-AACCCGGGACTTGGAGAAGCACTGC-3′. Real-time PCR was performed on an ABI PRISM 7700 system (Applied Biosystems) using the QuantiTect Probe PCR protocol (Qiagen). The rRNA Control kit (Applied Biosystems) was used to normalize transcript levels between samples.

### Isolation of Skp2 cDNA

A Skp2 cDNA was isolated by PCR amplification from an LNCaP 104-R1 Lambda ZAP-II cDNA library using the following primers derived from the Skp2 sequence: 5′-CAGCTCTGCAAGTTTAATGC-3′ and 5′-AAGAAGAGACACCATCCTGC-3′. The following program was used: pre-amplification at 94°C 5 min, 55°C 2 min, 30 cycles of 72°C 2 min, 94°C 0.5 min, 55°C 0.5 min, followed by 7 min at 72°C. Pfu (Stratagene) was used as DNA polymerase and dimethyl sulfoxide at 10% final concentration was used in the amplification reaction. A 1345 bp amplification product was inserted into EcoRV-digested pBluescript II vector for automated dideoxy sequence analysis. One Skp2 clone was chosen for sequence verification and was used in all subsequent experiments. The Skp2 cDNA was transferred from this pBluescript clone to the pMV7 retroviral vector for constitutive expression in LNCaP 104-R1 cells.

### Stable retroviral infection of Skp2

104-R1 cells were infected with pMV7 retrovirus containing Skp2 inserts that was generated in ΦNX-Ampho packaging cells using procedures described previously [10]. The ΦNX-Ampho packaging cell line was provided by Garry Nolan of Stanford University. Stably infected cells were selected by G418.

### Expression of GST-Skp2 protein

SmaI-HindIII fragments of Skp2 cDNAs were inserted into SmaI-HindIII-cut pGEX-KG [40]. The plasmids were transfected into E. coli BL-21-CodonPlus-RIL cells (Stratagene) for isopropyl thiogalactoside-induced expression of glutathione sulfur transferase (GST)-Skp2 fusion proteins.

### *In Vitro* assay of Cdk2 activity

Cell lysates were made from LNCaP 104-R1 cells infected with MV7 empty virus and LNCaP 104-R1 cells overexpressing Skp2 grown for 3 days in the presence or absence of 10 nM R1881. Assay of Cdk2 activity using histone H1 as substrate was described previously [10]. For Cdk2 phosphorylation of a synthetic Skp2 peptide, two 12 residue peptides (GHPESPPRKRLK and GHPEAPPRKRLK) corresponding to positions 60 to 71 of the wild-type Skp2 protein and was used as substrates in kinase reactions with immunoprecipitated Cdk2. Cell lysates were made from LNCaP 104-R1 cells grown for 4 days in the presence or absence of 10 nM R1881. Aliquots of lysate (1 mg total protein) prepared as described previously [10] were incubated with 2 mg Cdk2 antibody bound to protein G-agarose beads. After washing 3 times in lysis buffer and 2 times in kinase buffer (50 mM HEPES pH 7.5; 10 mM MgCl2, 0.5 mM DTT and 0.02% Triton X-100), beads were incubated with 10 mCi [g-32P]ATP and 20 mM peptide in a total volume of 25 ml for 30 min at 30°C. Reactions were terminated by the addition of 3 ml 0.5 M EDTA and 10 ml aliquots were spotted on 2.5 cm P-81 filter discs (Whatman). Discs were washed 5 times with 0.5% H3PO4 and once with 50% ethanol/0.05% H3PO4 to remove unincorporated ATP. Incorporated label was determined by liquid scintillation spectrophotometry. Blanks consisted of kinase reactions using antibody-loaded agarose beads not incubated with cell lysate. Reactions were carried out in quadruplicate.

### AR and Skp2 overexpression in PC-3 cells

PC-3 cells were transfected with LNCX-2 plasmid containing wild-type human AR or LNCaP cells' mutant AR cDNA and selected with neomycin G418 as previously described [32]. PC-3 cells overexpressing wild type AR or LNCaP mutant AR were then denoted as PC-3^AR^ or PC-3^LNCaP-AR^. PC-3^AR^ cells further transfected with LPCX plasmid containing Skp2 cDNA or LPCX control plasmid were selected with puromycin. Antibiotic-resistant colonies were expanded and screened for increased target protein expression by Western blot analysis.

### Public domain data

Expression profiles of selective genes from datasets containing tumor and adjacent normal tissues from PCa patients, including GSE6919 [41], which contains 23 normal prostate and 89 prostate carcinoma tissues, and Singh prostate datasets [42], which contains 50 normal prostate gland samples and 52 prostate carcinoma samples, were downloaded from Oncomine (http://www.oncomine.com) without further processing.

### Data Analysis

Data are presented as the mean +/− SD of at least three experiments or are representative of experiments repeated at least three times. Student's t test (two-tailed, paired) was used to evaluate the statistical significance of results from proliferation assay experiments.

## Results

### Androgen treatment suppressed proliferation of AR-rich CRPC cells

**Figure 1:**
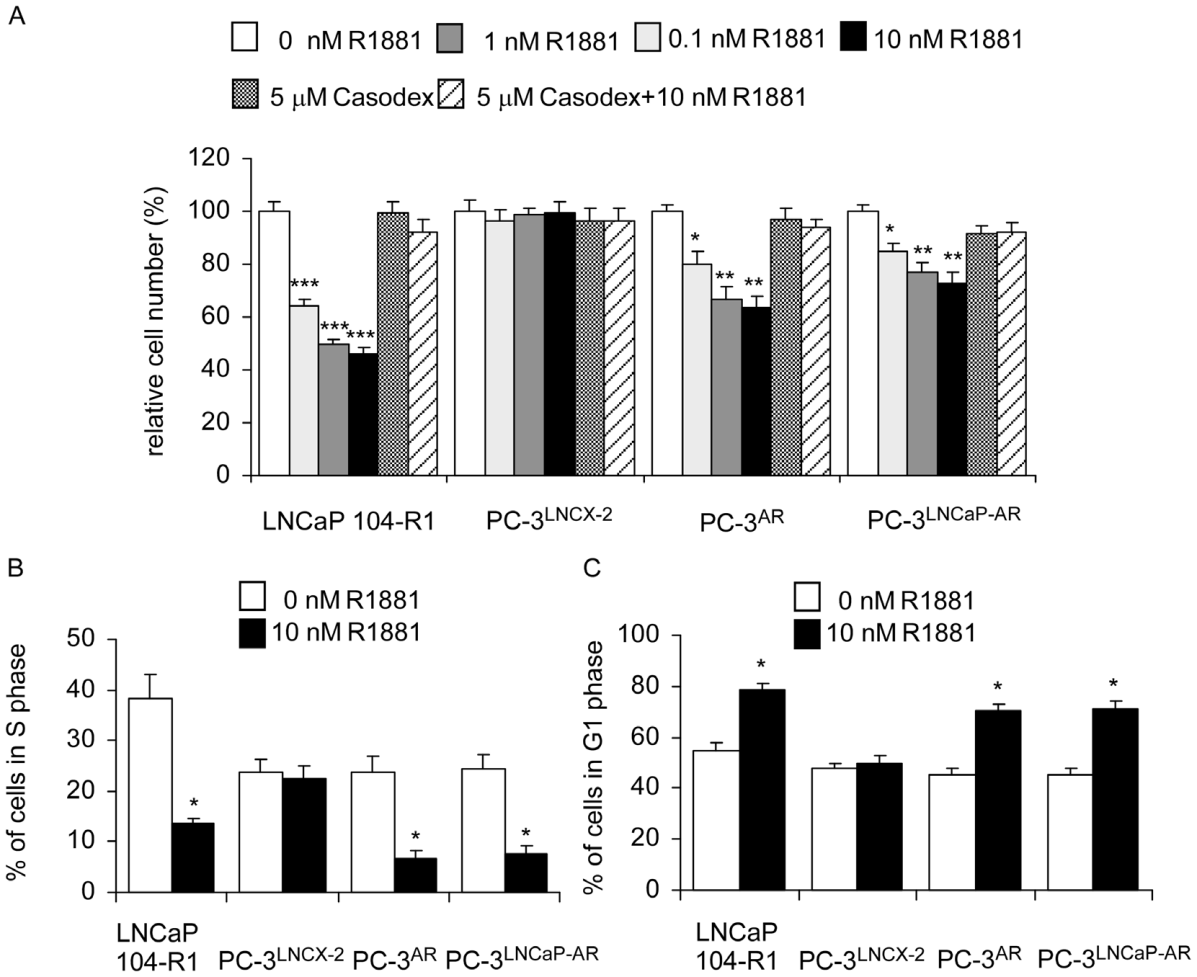
Effect of androgen on cell proliferation and cell cycle in LNCaP 104-R1 cells and PC-3 sublines. (A) LNCaP 104-R1 cells, PC-3 cells with control plasmid LNCX-2 (PC-3^LNCX-2^), PC-3 cells overexpressing wild type AR (PC-3^AR^), and PC-3 cells overexpressing LNCaP AR (PC-3^LNCaP-AR^) were treated with increasing concentration of synthetic androgen R1881 or antiandrogen Casodex for 96 hrs. Relative cell number was determined by fluorometric DNA assay described in Materials and Methods and was normalized to cell number of the control cells (no treatment). LNCaP 104-R1, PC-3^LNCX-2^, PC-3^AR^, and PC-3^LNCaP-AR^ cells were treated with or without 10 nM R1881 for 96 h. Percentage of cell population of LNCaP 104-R1, PC-3^LNCX-2^, PC-3^AR^, and PC-3^LNCaP-AR^ cells in S phase (B) and G1 phase (C) was determined by PI-staining flow cytometry. Values represent the mean +/− Standard Error derived from 5 independent experiments. Asterisk *, **, and *** denote significant difference *p*<0.05, *p*<0.01, *p*<0.001, respectively, of the treated cells as compared to control cells.

Treatment with synthetic androgen R1881 dose-dependently suppressed cell proliferation of AR-rich LNCaP 104-R1, PC-3 overexpressing wild type AR (PC-3^AR^), and PC-3 overexpressing LNCaP mutant AR (PC-3^LNCaPAR^) cells but not control AR-negative PC-3 cells (PC-3^LNCX-2^) (Fig. 1A). Antiandrogen Casodex blocked the androgenic suppression, confirming that androgenic inhibition on cell proliferation was through AR (Fig. 1A). Treatment with 10 nM R1881 decreased percentage of cell population in S phase and induced G1 phase cell cycle arrest in LNCaP 104-R1, PC-3^AR^, and PC-3^LNCaPAR^ cells but not control PC-3^LNCX-2^ cells (Fig. 1B, 1C).

### Androgen treatment affects cell cycle regulating proteins in CRPC cells

**Figure 2:**
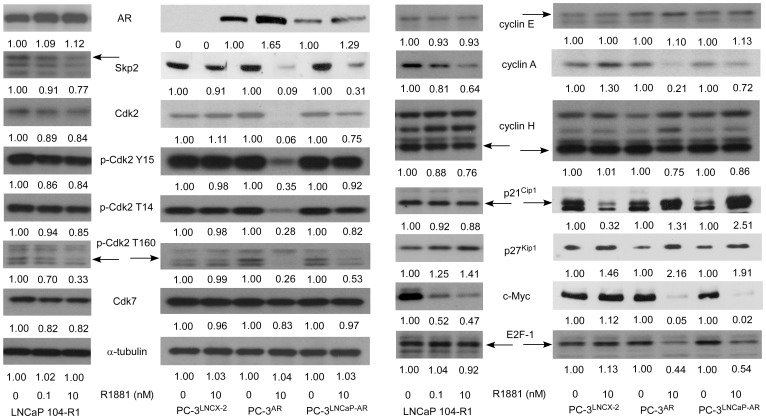
Effects of androgen on cell cycle-regulatory proteins in LNCaP 104-R1, PC-3^LNCX-2^, PC-3^AR^, and PC-3^LNCaP-AR^ cells. Protein expression of AR, Skp2, Cdk2, phospho-Cdk2 Tyr15, phospho-Cdk2 Thr14, phospho-Cdk2 Thr160, Cdk7, cyclin E, cyclinA, cyclin H, p21^cip^, p27^Kip^, c-Myc, and E2F-1 was determined by Western blotting assay in LNCaP 104-R1, PC-3^LNCX-2^, PC-3^AR^, and PC-3^LNCaP-AR^ cells treated with different concentration of R1881 for 96 h. Protein abundance of α-tubulin was used as loading control.

**Figure 3:**
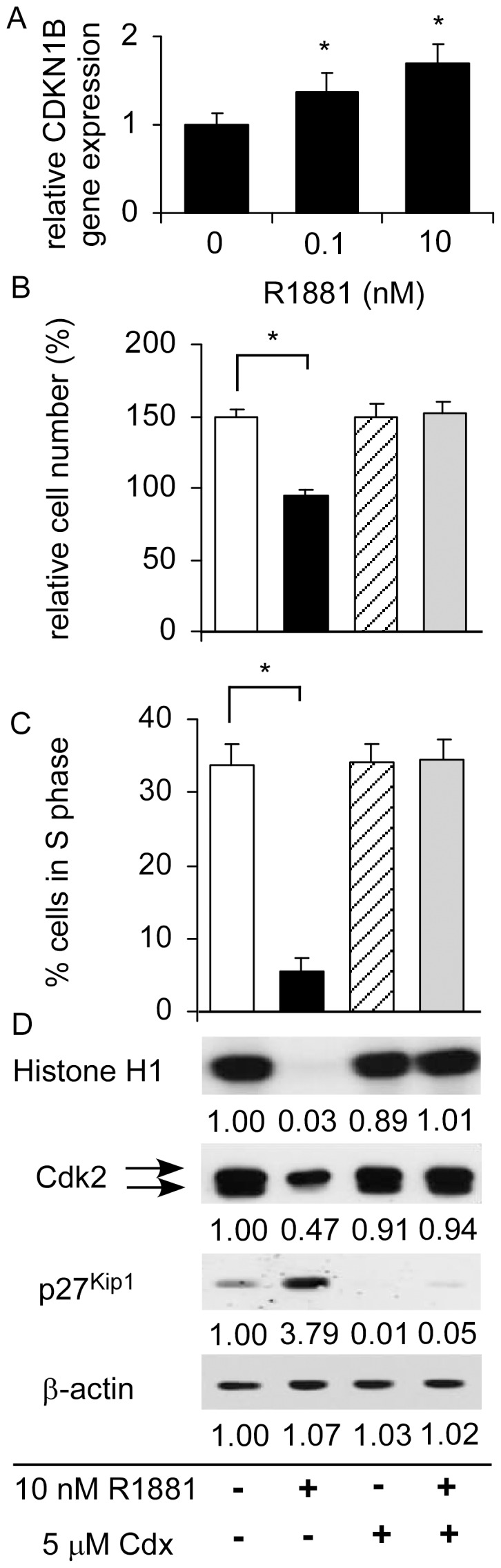
Effects of androgen and anti-androgen on cell proliferation, cell cycle, and Cdk2 activity in LNCaP 104-R1 cells. (A) Gene expression of CDKN1B was determined in LNCaP 104-R1 cells treated with 0, 0.1, or 10 nM R1881 for 96 h using qRT-PCR. LNCaP 104-R1 cells treated with 10 nm R1881 in the presence or absence of 5 µm anti-androgen Casodex for 96 h. Asterisk * denotes significant difference *p*<0.05 of the treated cells as compared to control cells. (B) Relative cell number was determined by fluorometric DNA assay. (C) Percentage of cell population in S phase was determined by flow cytometry analysis. Asterisk * denotes a significant difference (*p*<0.05) of the treated cells as compared to control cells. (D) Histone H1 phosphorylation was assayed using Cdk2 immunoprecipitated from cell lysate containing 2 mg protein. Relative radioactivity was determined by scanning with a Storm 860 phosphoimager (Molecular Dynamics, Sunnyvale, CA, U.S.A.). Protein expression level of Cdk2, p27^Kip1^, and β-actin were determined by Western blotting from the same cell lysates. Abundance of β-actin protein was used as loading control.

Androgen treatment slightly increased AR expression but dramatically increased cell cycle inhibitor p27^Kip1^ in LNCaP 104-R1, PC-3^AR^, and PC-3^LNCaPAR^ cells (Fig. 2). In the opposite, androgen treatment decreased protein expression of Skp2, Cdk2, phospho-Cdk2 Tyr15, phospho-Cdk2 Thr14, phospho-Cdk2 Thr160, Cdk7, cyclin A, cyclin H, and c-Myc in LNCaP 104-R1, PC-3^AR^, and PC-3^LNCaPAR^ cells (Fig. 2). Protein level of p27^Kip1^ was inverse-correlated to Skp2 in these CRPC cells, which was consistent with the fact that Skp2 targets p27^Kip1^ for ubiquitination and degradation [18]–[20]. Abundance of p21^Cip1^ was slightly decreased in LNCaP 104-R1 cells but was increased in PC-3^AR^ and PC-3^LNCaPAR^ cells by androgen. Abundance of E2F-1 was slightly decreased in LNCaP 104-R1 cells but was dramatically reduced in PC-3^AR^ and PC-3^LNCaPAR^ cells by androgen. Abundance of cyclin E was not significantly affected by androgen treatment. Activation of Cdk2 complexes requires dephosphorylation of Thr14 and Tyr15 on Cdk2 by cdc25 phosphatase and phosphorylation of Thr160 on Cdk2 [26], [27], which is mediated by CAK, a complex of Cdk7 and cyclin H [28]. Although phosphorylation of Thr14 and Tyr15 was slightly decreased by androgen treatment, phosphorylation of Thr160 was dramatically suppressed by androgen treatment, possibly due to the reduction of Cdk7 and cyclin H protein expression (Fig. 2). Androgen treatment increased p27^Kip1^, cyclin A, and c-Myc while decreased p21^Cip1^ and Skp2 in control AR-negative PC-3^LNCX-2^ cells. Since androgen did not affect the proliferation and cell cycle progression of PC-3^LNCX-2^ cells, the roles of these proteins in PC-3^LNCX-2^ cells was not clear. Skp2 targets p27^Kip1^ for ubiquitination and degradation [18]–[20]. However, under treatment of 0.1 and 10 nM R1881, gene expression level of CDKN1B (p27^Kip1^) increased 1.3 and 1.7 fold, respectively (Fig. 3A). As c-Myc has been reported to repress FOXO3a-mediated transcription of CDKN1B [43], reduction of c-Myc caused by androgen treatment may contributed to the increase of CDKN1B gene expression (Fig. 2). We therefore believe that reduction of protein degradation and increase of gene transcription both contributed to the increase of p27^Kip1^ protein level, which may therefore induce G1 cell cycle arrest in CRPC cells.

### Androgen treatment suppressed Cdk2 activity

Cdk2 is a histone H1 kinase responsible for the phosphorylation of histone during the cell cycle transition from G1 to S phase [44]–[46]. In order to confirm that Cdk2 activity was suppressed by androgen treatment, we used histone H1 as substrate for the kinase activity assay. Reduction of cell proliferation (Fig. 3B) and decrease in S phase cell population (Fig. 3C) in LNCaP 104-R1 cells caused by androgen treatment were closely associated with the decline of Cdk2 activity as detected by the reduction in the phosphorylation of histone H1, the lessening of a faster-migrating form of Cdk2, and an increase of the Cdk inhibitor p27^Kip1^ abundance (Fig. 3D). The faster-migrating Cdk2 was identified previously as an active form of Cdk2 that is phosphorylated at Thr160 [47]. Antiandrogen Casodex treatment blocked the effects of androgen on cell proliferation, cell cycle, phosphorylation of histone H1, and activity of Cdk2 (Fig. 3B, C, D).

### Overexpression of Skp2 blocked androgenic suppression in LNCaP 104-R1 cells

**Figure 4:**
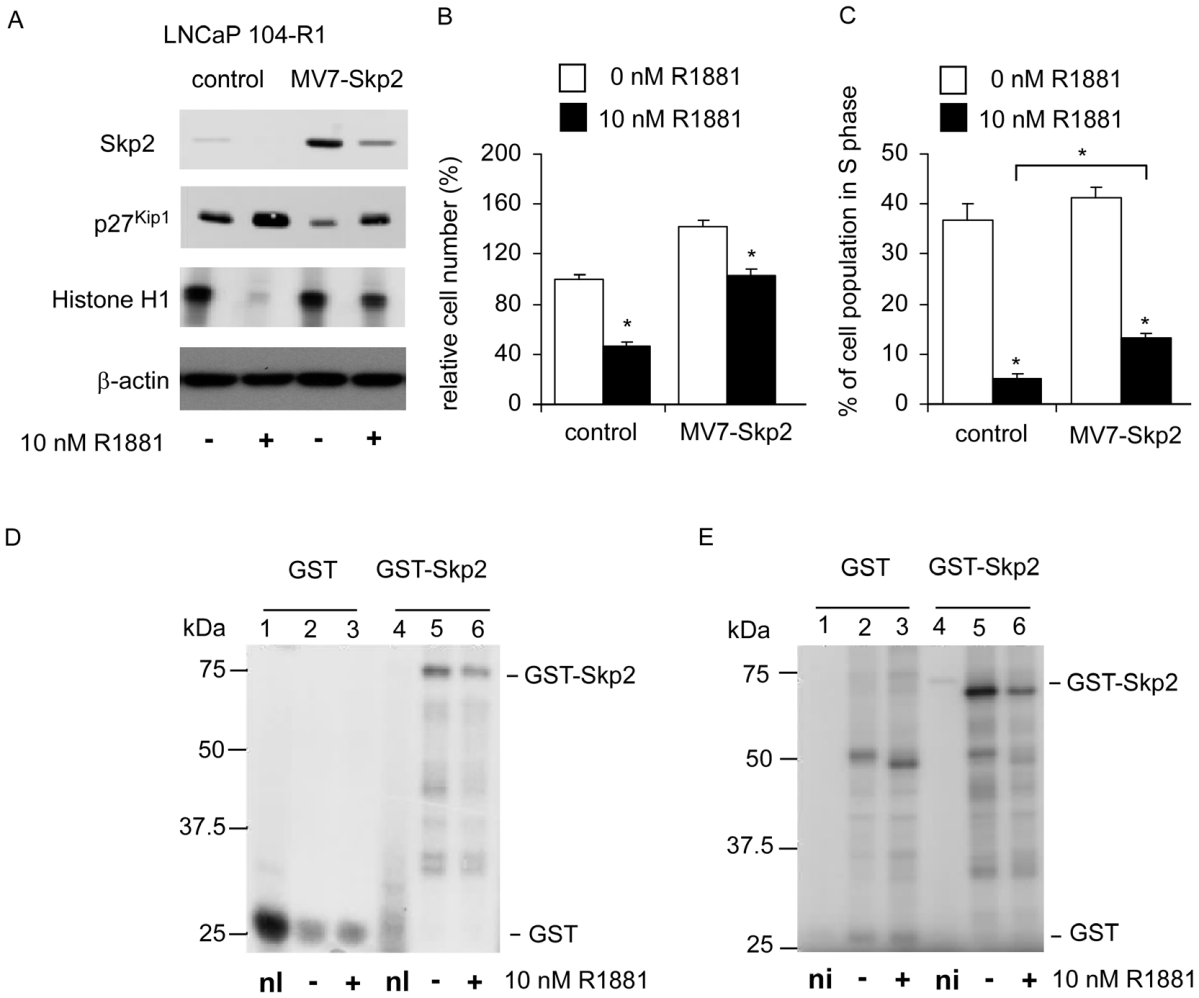
Effects of overexpression of Skp2 on proliferation, cell cycle, Skp2 phosphorylation, and Cdk2 activity in LNCaP 104-R1 cells under androgen treatment. (A) Protein expression level of Skp2 and p27^Kip1^ in LNCaP 104-R1 cells infected with pMV7 empty retrovirus (control) or pMV7 retrovirus containing Skp2 was determined by Western blotting. Cells were grown for 3 days in the presence or absence of 10 nm R1881. Cdk2 activity was measured by *in vitro* phosphorylation of histone H1 using Cdk2 immunoprecipitates. Abundance of β-actin protein was used as loading control. (B) Cell proliferation of LNCaP 104-R1 cells infected with pMV7 empty retrovirus (control) or pMV7 retrovirus containing Skp2 treated with or without 10 nM R1881 for 96 hours was determined using the fluorometric DNA assay and was normalized to the cell number of control group (no treatment). (C) Percentage of LNCaP 104-R1 cells in S phase was determined by flow cytometry. Cells were grown for 3 days in the presence or absence of 10 nm R1881. Asterisk denotes a significant difference (*p*<0.05) from control cells. (D) LNCaP 104-R1 cells were treated with or without 10 nM R1881 for 3 days. Fusion proteins of control GST (lanes 1–3) and bacterially-expressed GST-Skp2 (lanes 4–6) bound to glutathione-agarose beads was incubated with 200 µg whole cell lysates from untreated and androgen-treated 104-R1 cells in the presence of 10 µCi [^32^P]-ATP for 30 min at room temperature. “nl” represents as no lysate. Phosphorylated proteins were eluted in Laemmli gel loading buffer and separated on a 10% SDS-PAGE gel that was then dried and exposed to Kodak X OMAT AR film for 3 days. (E) 104-R1 cells were treated with or without 10 nM R1881 for 3 days. Phosphorylation of eluted GST (lanes 1–3) and GST-Skp2 (lanes 4–6) fusion proteins was determined by immunoprecipitated Cdk2. Cdk2 was immunoprecipitated from aliquots of lysate containing 1 mg protein prepared from untreated (lanes 2, 5 and 8) or R1881-treated (lanes 3, 6 and 9) 104-R1 cells. Control lanes (lanes 1, 4, and 7) labeled “ni” indicated no immunoprecipitated Cdk2. Cdk2 activity was determined by methods described in Materials and Methods.

Skp2 is phosphorylated and activated by Cdk2 [20] as well as forms a stable complex with the cyclin A and Cdk2 [20]. We previously reported that overexpression of Skp2 partially blocked the proliferation of LNCaP 104-R2 cells [15]. In this study, we determined the relationship between overexpression Skp2 and Cdk2 activity. Overexpression of Skp2 in LNCaP 104-R1 cells relieved androgenic repression of Cdk2 activity as assayed of *in vitro* phosphorylation of histone H1 immunoprecipitated with Cdk2 (Fig. 4A). Measurement of kinase activity in Cdk4 immunoprecipitates prepared from these cells did not show difference (data not shown). Overexpression of Skp2 in LNCaP 104-R1 cells partially reduced the induction of p27^Kip1^ (Fig. 4A), growth inhibition (Fig. 4B), and G1 cell cycle arrest (Fig. 4C) caused by androgen treatment. The basal level of p27^Kip1^ in Skp2-overexpressed 104-R1 cells was much less compared to control 104-R1 cells (Fig. 4A). This may explain why the 104-R1 cells overexpressing Skp2 proliferated 1.42 fold faster than the control 104-R1 cells (Fig. 4B).

### Androgen regulates phosphorylation of Skp2 through Cdk2

Unfortunately, antibodies detecting the phosphorylation of Ser64 or Ser72 on Skp2 are not commercial available. To determine if androgen treatment affects the phosphorylation of Skp2, we incubated bacterially-expressed GST-Skp2 fusion proteins bound to glutathione-agarose beads with whole cell lysates from untreated and androgen-treated LNCaP 104-R1 cells in the presence of [g-^32^P]ATP. We found that lysate from androgen-treated 104-R1 cells contained less kinase activity capable of phosphorylating the 74 kDa GST-Skp2 fusion proteins as compared with the lysate from untreated cells (Fig. 4D). This result indicated that androgen treatment reduced the total phosphorylation on Skp2. We then investigated if Cdk2 actually phosphorylate Skp2. When GST-Skp2 fusion proteins were used as substrates in kinase reactions with Cdk2 immunoprecipitated from LNCaP 104-R1 cell lysates, similar results were observed as that from Fig. 4D (Fig. 4E). This result revealed that Cdk2 phosphorylated Skp2 and the activity of Cdk2 to phosphorylate Skp2 was suppressed by androgen treatment in LNCaP 104-R1 cells.

### Overexpression of Skp2 blocked androgenic suppression in PC-3^AR^ and PC-3^LNCaPAR^ cells

**Figure 5:**
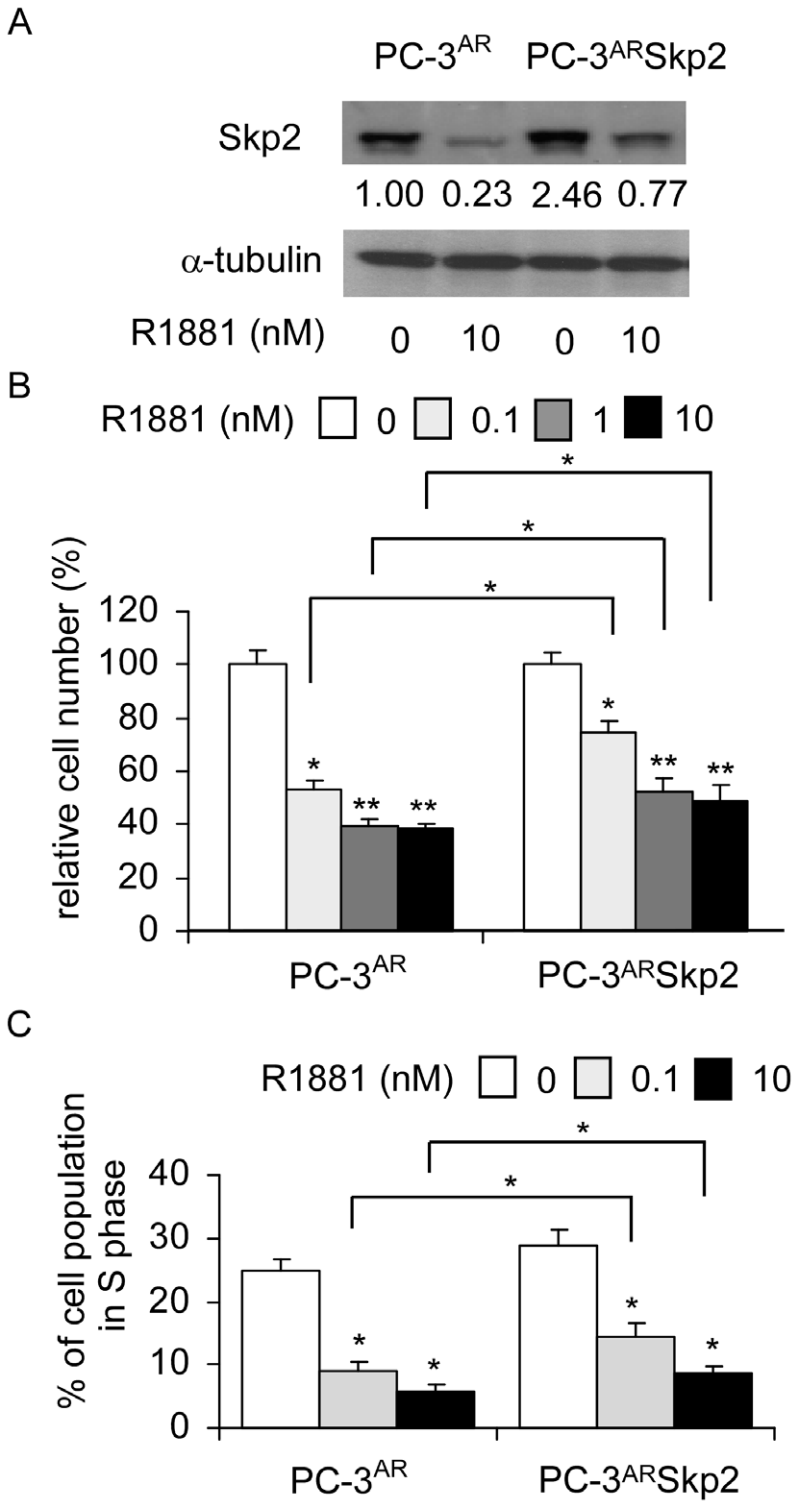
Androgenic response of PC-3 cells over-expressing AR and Skp2. (A) Protein expression of Skp2 was assayed by Western blotting in PC-3 cells re-expressing wild type AR (PC-3^AR^) cells and overexpressing either control vector or Skp2 in the absence or presence of 10 nM R1881 for 96 h. Effect of androgen on cell proliferation (B) or cell cycle progression (C) of these clones treated with increasing concentration of R1881 for 96 h was determined by 96-well proliferation assay and PI-staining flow cytometry analysis, respectively. Asterisks * and ** denote a significant difference *p*<0.05 and *p*<0.01, respectively, between the treatment and the control cells.

Similar to LNCaP 104-R1 cells, overexpression of Skp2 partially blocked the dose-dependent effects of androgenic inhibition on cell proliferation and cell cycle progression of PC-3^AR^ cells (Fig. 5).

### Correlation between Skp2, Cdk2, cyclin A, and Cdk7 in PCa tumors

**Figure 6:**
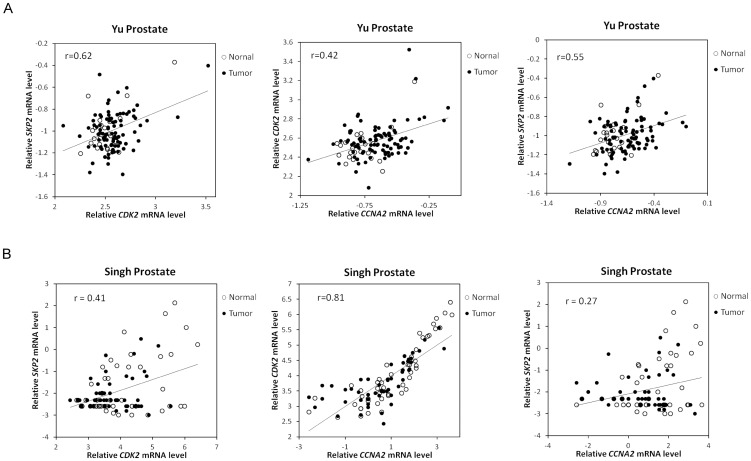
Positive correlation of gene expression levels of CCNA2, SKP2, and CDK2 in public domain datasets. Scatter plots showing correlation of indicated genes in GSE6919 (A) and Singh prostate datasets (B). The r value indicated correlation coefficient. Probe ID for CCNA2, CDK2, and SKP2 were 40697_at, 1792_g_at, and 1941_at in GSE6919, and 1943_at, 1792_at, and 39449_at in Singh prostate dataset.

**Figure 7:**
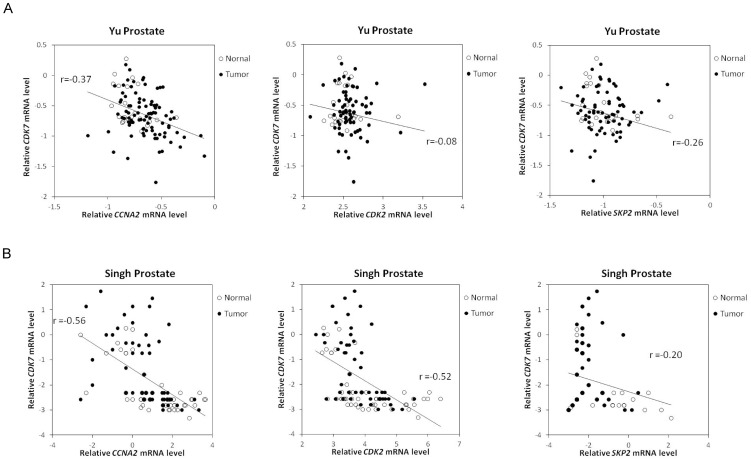
Negative correlation of gene expression levels of CDK7 with CCNA2, SKP2, and CDK2 in public domain datasets. Scatter plots showing correlation of indicated genes in GSE6919 (A) and Singh prostate datasets (B). The r value indicated correlation coefficient. Probe ID for Cdk7, CCNA2, CDK2, and SKP2 were 1969_s_at, 40697_at, 1792_g_at, and 1941_at in GSE6919, and 1969_s_at, 1943_at, 1792_at, and 39449_at in Singh prostate dataset.

According to the fact that expression and activity of Skp2 is regulated by Cdk2 and cyclin A in PCa cells and that Skp2, Cdk2, and cyclin A coordinately regulate the cellular proliferation in PCa cells, we hypothesized that gene expression level of Skp2 (SKP2), Cdk2 (CDK2), and cyclin A (CCNA2) may show good positive correlation in PCa tumors. Indeed, analysis of oncomine data indicated that gene expression level of SKP2, CDK2, and CCNA2 correlated positively well in both GSE6919 [41] (Fig. 6A) and Singh prostate tumor datasets [42] (Fig. 6B). As the phosphorylation of Thr160 on Cdk2 is mediated by Cdk7 [28], we determined if gene expression of CDK7 correlated to CDK2, SKP2, and CCNA2 in PCa tumors. Surprisingly, gene expression of CDK7 negatively correlated to CDK2, SKP2, and CCNA2 in both GSE6919 [41] (Fig. 7A) and Singh prostate tumor datasets [42] (Fig. 7B).

## Discussions

Androgen deprivation therapy is associated with several undesired side-effects, including sexual dysfunction, osteoporosis and bone fractures, hot flashes, fatigue, gynecomastia, anemia, depression, cognitive dysfunction, as well as increased risk of diabetes, coronary heart disease, myocardial infarction, sudden cardiac death, and stroke [7], [48]. A few clinical studies examined the possibility of using androgen for treatment of advanced PCa. Mathew reported that a PCa patient undergoing radical prostatectomy and LH-RH therapy maintained castrated levels of serum testosterone level and undetectable serum PSA for 15 years before serum PSA level elevated. The patient was then given testosterone to achieve a physiological level of testosterone. After an initial flare, the serum PSA level gradually declined for 18 months and did not relapse for 9 months. However, serum PSA levels dropped when testosterone supplement was discontinued [14]. Another clinical study investigated 15 PCa patients receiving androgen ablation in combination with anti-androgen therapy. These PCa patients were randomly separated into three groups, receiving 2.5, 5.0, or 7.5 mg transdermal testosterone per day, respectively. Serum testosterone level increased from castration levels to median concentrations of 305 ng/dl, 308 ng/dl, and 297 ng/dl, respectively. Only one patient experienced symptomatic progression while three patients showed dramatically decrease in PSA [49]. In another study, 5 patients received surgically castrated for at least 3 years developed advanced PCa and were given testosterone supplement [50]. For the first 12 months, all patients improved significantly without significant PSA increase [50]. After 18 months no side effects or metastasis were observed [50] and only one patient showed significant PSA elevation but was controlled by androgen withdrawal. Finally, 10 patients previously treated with radical prostatectomy for organ confined PCa with low serum total testosterone and symptoms of hypogonadism were treated with testosterone supplementation [51]. Serum total testosterone increased significantly from 197±67 to 591±180 ng/dl [51]. However, at a median follow up of 19 months, no patient had detectable PSA. These observations suggested that androgen may be a potential therapy for AR-positive CRPC PCa. We previously reported that androgen caused growth inhibition and G1 cell cycle arrest in LNCaP 104-R2 cells via suppression of c-Myc and Skp2 [15]. However, how androgen suppresses Skp2 in CRPC cells was not understood. In this study, we reported that androgen suppresses Skp2 activity and function via inhibition of protein abundance and activity of Cdk2 and cyclin A in LNCaP 104-R1 and PC-3^AR^ cells.

Cdk2 is an important component of the cell cycle machinery [26]. Complex of Cdk2-cyclin E is required for the transition of cells from G1 to S phase, while binding between Cdk2 and cyclin A is required to progress through the S phase [26]. Our observation indicated that although androgen treatment induced G1 cell cycle arrest, cyclin E protein level was not significantly affected by androgen treatment (Fig. 2). We therefore believe that complex of Cdk2-cyclin A may play important role in G1 cell cycle arrest induced by androgen treatment via regulation of Skp2 in CRPC cells. Activation of Cdk2 complexes requires dephosphorylation of Thr14 and Tyr15 on Cdk2 by cdc25 phosphatase and phosphorylation of Thr160 on Cdk2 [26], [27]. Phosphorylation of Thr160 on Cdk2 is mediated by Cdk7 and cyclin H [28]. We observed that, in CRPC cells, androgen treatment reduced protein abundance of Cdk2 (Fig. 2), phosphorylation of Thr160 on Cdk2 (Fig. 2), and kinase activity of Cdk2 on Skp2 proteins (Fig. 3, 4). The reduction of Thr160 on Cdk2 was possibly due to the androgenic suppression of Cdk7 and cyclin H (Fig. 2). Although androgen treatment also decreased phosphorylation of Thr14 and Tyr15 on Cdk2 in CRPC cells (Fig. 2), Cdk2 activity was inhibited by androgen treatment (Fig. 3), indicating that Thr160 played more important role in regulating the activity of Cdk2 in CRPC cells.

Androgen treatment also decreased protein abundance of cyclin A (Fig. 2). Cyclin A is a member of the cyclin family regulating cell cycle progression. There are two isoforms of cyclin A in human, the embryonic-specific form A1 and the somatic form A2. Cyclin A1 is prevalently expressed during meiosis and early on in embryogenesis while cyclin A2 is expressed in dividing somatic cells. Transcription of cyclin A is tightly regulated by the transcription factor E2F in a negative feedback loop [29]. The E2F family plays a crucial role in the control of cell cycle and action of tumor suppressor proteins. E2F-1, a family of E2F, has been reported to interact with Skp2 [52] and cyclin A2 [53]. We observed that abundance of E2F-1 was suppressed by androgen in CRPC cells (Fig. 2).

Androgen treatment caused the decline in Skp2 protein abundance (Fig. 2), decreased in phosphorylation of Skp2 (Fig. 4), and accumulation of p27^Kip1^ (Fig. 2–4), which in turn induced G1 cell cycle arrest in CRPC cells (Fig. 1). Enforced overexpression of the Skp2 protein in LNCaP 104-R1 cells reduced accumulation of p27^Kip1^ and partially blocked androgenic repression of Cdk2 activity, cell proliferation, and cell cycle progression. This observation confirmed that Cdk2, Skp2, and p27^Kip1^ were involved in the G1 cell cycle arrest caused by androgen treatment. Recently, small molecule inhibitors selectively inhibiting SCF-Skp2-mediated p27 degradation have been identified [54]. As we showed that Skp2 plays important role in regulating cell proliferation and cell cycle progression of CRPC cells, these inhibitors may be potential treatment for CRPC patients.

Our analysis of on-line gene array data indicated that in normal prostate epithelial cells and PCa cells, the gene expression level of SKP2 positively correlated well with CDK2 and CCNA2 in both normal and cancerous prostate tissues. This observation suggested the possibility that Skp2, Cdk2, and cyclin A play essential roles in regulating cell proliferation of normal prostate tissues and prostate tumors. Although we showed that androgen treatment decreased protein level of Cdk7, Cdk2, phosphorylation of Cdk2, Skp2, and cyclin A (Fig. 2), analysis of public gene array database showed that CDK7 negatively correlated to CDK2, SKP2, and CCNA2 (Fig. 7). CDK7 is a cyclin-dependent kinase which is both a CDK-activating kinase that phosphorylates cell-cycle regulatory CDKs, and a component of the general transcription factor TFIIH, which phosphorylates the largest subunit of Pol II [55]. CDK7 is inhibited by the phosphorylation of cyclin H, which is regulated by CDK7 itself [56]. This feed back regulation of Cdk7 by itself may explain the negative correlation between gene expression of CDK7 and CDK2, SKP2, and CCNA2.

Previously, we reported that androgen treatment suppresses mRNA and protein of c-Myc in LNCaP 104-R1 cells, which happens within hours [9], [10]. This observation suggests the possibility that androgen treatment inhibits transcription of c-Myc. Recent study indicated that androgen treatment induced formation of AR/β-catenin/TCF-4 complexes, which suppress c-Myc transcription [57]. The fact that overexpression of Skp2 can not completely blocked the suppressive effect of androgen in AR-positive CRPC cells (Fig. 4 and Fig. 5) confirmed that c-Myc also plays important role in regulation of androgenic suppression in CRPC cells. The c-Myc is a transcriptional factor and a well known oncogene. The c-Myc is activated by various mitogenic signal pathways, including Wnt, Shh and EGF. The c-Myc regulates cell proliferation, apoptosis, differentiation, and stem cell self-renewal. The c-Myc protein also contributes to metabolic adaptations, especially the Warburg effect, at many of the same steps in metabolic pathways as the AR in PCa cells [58] and regulates many glycolytic enzymes in cancer cells [58]–[60]. The c-Myc is essential for driving enhanced glutaminolysis in cancer cells and thus maintains the mitochondrial function and oxidative phospshorylation [58] as well as directly or indirectly (via miRNAs) regulates the glutamine transporters and mitochondrial glutaminase, GLS1 [61], [62]. AR signaling induced by androgen treatment suppresses cell proliferation of normal human prostate epithelial via AR/β-catenin/TCF-4 complex inhibition of c-Myc transcription [57]. A gain of function in c-Myc gene regulation is reported to be important for the conversion of AR signaling from a growth suppressor in normal prostate epithelial cells to an oncogene in PCa cells [63]. The expression of c-Myc is significantly elevated in androgen-independent LNCaP cells as compared to their parental androgen-dependent LNCaP cells [13], suggesting that metabolic pathways may be different in CRPC LNCaP cells as compared to the parental androgen-dependent LNCaP cells. Androgen treatment increased c-Myc in androgen-dependent LNCaP 104-S cells, promoted the cell cycle progression, and therefore stimulated the cell proliferation [7], [9]–[11], [13], [15]. On the other hand, androgen treatment suppressed c-Myc expression, induced G1 cell cycle arrest, and thus inhibited cell growth in androgen-independent LNCaP cells [7], [9]–[11], [13], [15]. Overexpression of c-Myc partially blocked the G1 cell cycle arrest induced by androgen [10], [15]. Skp2 and c-Myc may be two important therapeutic targets for patient with CRPC.

In conclusion, our study indicated that androgen treatment suppresses the proliferation and cell cycle progression of CRPC cells partially through inhibition of the function and activity of Cyclin A, Cdk2, and Skp2. This study could benefit patients with castration resistant prostate cancer by targeting Skp2, Cdk2, and cyclin A using androgen treatment.
