# Correction: Androgen Suppresses the Proliferation of Androgen Receptor-Positive Castration-Resistant Prostate Cancer Cells via Inhibition of Cdk2, CyclinA, and Skp2

**DOI:** 10.1371/journal.pone.0111731

**Published:** 2014-10-16

**Authors:** The PLOS ONE Staff

There are errors in the author affiliations for Chung-Ho Chang and Chih-Pin Chuu. The correct author affiliations are shown here:

John M. Kokontis^1☯^, Hui-Ping Lin^2☯^, Shih Sheng Jiang^2^, Ching-Yu Lin^3^, Junichi Fukuchi^1,4^, Richard A. Hiipakka^1^, Chi-Jung Chung^5^, Tzu-Min Chan^6,7^, Shutsung Liao^1^, Chung-Ho Chang^3^, Chih-Pin Chuu^3,8,9,10*^

^1^The Ben May Department for Cancer Research, The University of Chicago, 929 East 57th Street, Chicago, Illinois 60637, U.S.A. ^2^National Institute of Cancer Research, National Health Research Institutes, Miaoli County, Taiwan ^3^Institute of Cellular and System Medicine, National Health Research Institutes, Miaoli, Taiwan ^4^Pharmaceuticals and Medical Devises Agency, Tokyo, Japan ^5^Department of Health Risk Management, China Medical University, Taichung City, Taiwan ^6^Department of Medical Education and Research, China Medical University Beigang Hospital, Yunlin, Taiwan ^7^Department of Medical Education and Research, Tainan Municipal An-Nan Hospital-China Medical University, Tainan, Taiwan ^8^Graduate Institute of Basic Medical Science, China Medical University, Taichung City, Taiwan ^9^Biotechnology Center, National Chung Hsing University, Taichung, Taiwan, Taichung City, Taiwan ^10^Ph.D. program in Environmental and Occupational Medicine, Kaohsiung Medical University, Kaohsiung City, Taiwan

* Email: cpchuu@nhri.org.tw

 These authors contributed equally to this work.
